# Follow-up Study of Microflora Changes in Crevicular Gingival Fluid in Obese Subjects After Bariatric Surgery

**DOI:** 10.1007/s11695-020-05006-0

**Published:** 2020-09-30

**Authors:** Bettina Balogh, Sándor Somodi, Miklós Tanyi, Cecília Miszti, Ildikó Márton, Barna Kelentey

**Affiliations:** 1 Department of Restorative Dentistry, Faculty of Dentistry, University of Debrecen, Nagyerdei krt. 98, Debrecen, H-4032 Hungary; 2 Department of Internal Medicine, Faculty of Medicine, University of Debrecen, Debrecen, Hungary; 3 Department of Surgery, Faculty of Medicine, University of Debrecen, Debrecen, Hungary; 4 Department of Medical Microbiology, Faculty of Medicine, University of Debrecen, Debrecen, Hungary

**Keywords:** Bariatric surgery, Obesity, Crevicular gingival fluid, Non-albicans *Candida* species

## Abstract

The objective of our study was to investigate the effect of weight loss on the crevicular microflora following bariatric surgery. Crevicular fluid samples were taken from 57 subjects: 22 were in the normal control group; 18 in the obese control group; and 17 patients had had bariatric surgery, who underwent a repeat sampling 6 to 12 months after the operation. Crevicular fluid samples were analyzed by MALDI-TOF MS analysis. After surgery and weight loss, the mean germ count increased, albeit not significantly. Also, *Candida albicans* and non-albicans *Candida* species: *C. dubliniensis*, *C. kefyr*, and *C. lusitaniae* appeared after surgery (*p* < 0.05) in subjects where *Neisseria* was either absent throughout or eliminated after surgery. However, periodontitis did not develop during this time in our subjects.

## Introduction

Overweight and obesity are ever more common these days, especially in developed countries. The reason why obesity is important in dentistry is because many studies indicate a link between body weight and certain dental diseases. There is evidence that intense adipose tissue accumulation may cause oral inflammatory diseases because hyper-inflammatory periodontal tissues react with an accentuated response to infection by periodontal pathogens and therefore, the incidence of periodontitis in the obese is greater than in the average population [1].

Periodontitis is a multifactorial inflammatory disease which involves continuous destruction of tissues surrounding and supporting the teeth. The role of bacteria in the onset of periodontitis is essential; however, the development, clinical appearance, and level of progression of the disease are influenced by host-related factors. Examples of conditions associated with an increased risk of developing periodontitis include diabetes, smoking, elderly age, and stress [2, 3]. Relative to bacteria, fungi such as various *Candida* species make up a minor proportion of the oral and crevicular fluid flora. However, their numbers may increase in conditions of nutrient deficiency or reduced immune system function; examples of other factors predisposing to Candida growth include removable denture use, diabetes, and limited saliva production [4, 5].

Healthy diet and exercise are an essential part of obesity treatment. If these fail to produce results, it is possible to perform body weight reduction surgery, especially when treating markedly severe obesity and its concomitant diseases (e.g., type 2 diabetes, hyperlipidemia, hypertension) [6]. Several previous studies indicate that despite significant weight loss after surgery, the condition of periodontal tissues and the composition of crevicular fluid fail to improve; some studies have even found a deterioration in these outcomes [7, 8].

In our study, we aimed to investigate the differences in crevicular gingival fluid microflora between patients in the obese group and the average (normal body weight) population, as well as to assess the effect of weight loss after bariatric surgery on the crevicular microflora.

## Material and Methods

### Study Sample

We investigated a total of 57 individuals, 33 female and 24 male patients, in the age bracket 18 to 58 years. In the first study group, we included 22 healthy, normal body weight control subjects from persons visiting the Faculty of Dentistry at the University of Debrecen (UD). In the second group, we investigated 35 patients with obesity visiting the bariatric outpatient office at UD’s Clinical Department of Internal Medicine or the Department of Surgery for treatment. Of these subjects, 17 underwent repeat sampling an average of 6 to 12 months after weight reduction surgery (gastric bypass). Exclusion criteria included full or partial denture use, presence of any chronic systemic disease (e.g., diabetes) or autoimmune disease, use of antibiotics within the last 3 months, use of steroids or drugs with side effects manifesting as reduced saliva production regularly or within the last 3 months, pregnancy, as well as smoking (current or past), poor oral hygiene, and age over 60 since all these factors predispose to periodontal disease and/or *Candida* proliferation [5].

Before starting their participation in the research, subjects were given verbal information and signed a statement of consent detailing the course and purpose of the study. The study was approved by the National Ethics Committee (ethics approval Nr. ETT TUKEB 12622-8/2015/EKU) and carried out in accordance with the Declaration of Helsinki.

### Sample Collection and Analysis

We ran microbiological tests on fluid collected from the gingival crevice (groove between the tooth and free gingiva) and repeated them in patients with obesity about 6 to 12 months after surgery. Using sterile absorbent paper points, we collected samples from the crevices of the canines and first molars. Sampling was always carried out by the same dentist, from 3 different locations per patient and procedure. Samples were always collected at the same time of day. The area was isolated (wiped with a sterile gauze pad) and sterile paper points were inserted with their ends into the crevice with a pair of dental tweezers until resistance was first felt and held for 5 s to absorb as much crevicular fluid as possible. (We made sure to avoid points being contaminated by saliva in the oral cavity.) During this time, a sterilized Eppendorf centrifuge tube containing 1 mL of physiological saline was kept standing by with its cap on. We removed the absorbent points with tweezers and tossed them into the tube. The labeled test tubes were then immediately transferred to the Department of Medical Microbiology, where the samples were processed by standard laboratory methods. Clinical specimens were inoculated onto blood agar, chocolate agar, and Sabouraud dextrose agar plates and were grown overnight at 37 °C. Identification was performed by matrix-assisted laser desorption ionization–time of flight mass spectrometry (MALDI-TOF MS) and MALDI Biotyper 3.0 software with Bruker taxonomy library (Bruker Daltonic) [9].

We also assessed periodontal status by measuring sulcus or, in a pathological case, pocket depth (distance between the bottom of the gingival crevice and free gingival margin) and, if present, loss of periodontal attachment (distance between crown enamel–root cement junction and bottom of gingival crevice). This was performed using a Williams periodontal probe. Periodontitis was defined as 2 or more teeth with a loss of attachment ≥ 3 mm on the buccal or oral surface and a periodontal pocket deeper than 3 mm [3].

### Statistical Analysis

Variables were described using absolute frequencies for categorical variables and counts, arithmetic means, and standard deviations (SD) for continuous variables. Follow-up versus baseline comparisons were based on Wilcoxon’s matched-pairs signed-ranks test for categorical and Student’s paired *t* test (if distributional assumptions were satisfied) or Wilcoxon’s matched-pairs signed-ranks test (otherwise) for continuous variables. For between-groups comparisons, Student’s two-sample *t* test or Wilcoxon’s rank-sum test was used, subject to distributional assumptions being satisfied; the necessity of adjustment for age or sex was assessed using multiple linear regression. The significance criterion was set to *α* = 0.05. The statistical package Stata (Release 15, StataCorp, College Station, USA) was used for data handling and analysis.

## Results

Of the total of 57 subjects in the study, 22 patients—13 women and 9 men—were in the normal control group, with a mean age of 33.9 (SD 9.24) years, ranging from 18 to 53 years and a mean body mass index (BMI) of 23.3 (SD 2.16) kg/m^2^. Of the 35 obese subjects, 18 did not undergo surgery and had no change to their body weight; these 13 women and 5 men made up the obese control group, with mean age 44.1 (SD 10.79) years, ranging from 19 to 58 years and mean BMI 44.5 (SD 6.52) kg/m^2^. Seventeen of the obese subjects underwent weight loss surgery (surgical patient group); the mean age of these 7 women and 10 men was 39.4 (SD 10.17) years, ranging from 21 to 54 years, and their mean BMI before surgery was 46 (SD 7.03) kg/m^2^. Repeat sampling was performed an average 11.3 months after gastric bypass surgery, at which time a mean weight loss of 41.5 kg and a new mean BMI of 31.5 (SD 8.3) kg/m^2^ was recorded (Table 1). There was no loss to follow-up in the surgery group.

**Table 1:** Descriptive statistics of age, gender, and BMI by study group

	Normal controls (*n* = 22)	Obese controls (*n* = 18)	Surgery patients (*n* = 17)
Age	33.9 (9.24) [18–53]	44.1 (10.79) [19–58]^§^	39.4 (10.17) [21–54]
Male/female	9/13	5/13	10/7
BMI at baseline	23.3 (2.16) [19.8–29.5]	44.5 (6.52) [35.0–58.0]^§^	46.0 (7.03) [34.5–60.0]^§^
BMI at follow-up	N/A	N/A	31.5 (8.3)*
Change in BMI	N/A	N/A	− 14.5 (4.45)

Values are count/count or mean (SD) [minimum-maximum]

*p < 0.0001 versus baseline

^§^*p* < 0.01 versus normal controls

As to periodontal conditions, no signs of inflammation were observed in any group, and no attachment loss greater than 3 mm or pathologically deep periodontal pockets were detected, including in subjects after surgery and weight loss.

Crevicular fluid samples included *Actinomyces*, *Candida*, *Capnocytophaga*, *Eikenella*, *Fusobacterium*, *Granulicatella*, *Haemophilus*, *Lachnoanaerobaculum*, *Lactobacillus*, *Micrococcus*, *Neisseria*, *Prevotella*, *Rothia*, *Staphylococcus*, *Streptococcus*, and *Veillonella* genera, as detailed in Table 2. Adjustment for age or sex proved unnecessary when comparing the study groups in terms of germ counts at baseline.

**Table 2:** Presence of bacterial genera in crevicular fluid samples by study group

	Normal controls (*n* = 22)	Obese controls (*n* = 18)	Preoperative (*n* = 17)	Postoperative (*n* = 17)
*Actinomyces*	1; 4.545	0; 0	1; 1.882	0; 0
*Candida*	1; 454.5	2; 7.333	0; 0	5; 44,176^§^
*Capnocytophaga*	1; 4.545	2; 11.11	0; 0	0; 0
*Eikenella*	0; 0	0; 0	0; 0	1; 58.82
*Fusobacterium*	4; 140.9	3; 1167	2; 647.0	3; 176.4
*Granulicatella*	0; 0	0; 0	1; 588.2	0; 0
*Haemophilus*	1; 4.545	6; 629.5*	2; 70.58	4; 1824
*Lachnoanaerobaculum*	1; 4.545	0; 0	0; 0	0; 0
*Lactobacillus*	0; 0	1; 1.777	0; 0	0; 0
*Micrococcus*	0; 0	1; 5.555	0; 0	0; 0
*Neisseria*	1; 1.454	10; 11,906*	10; 1606*	4; 6718
*Prevotella*	3; 590.9	11; 6783*	7; 2529	11; 13,882
*Rothia*	0; 0	1; 5.555	1; 58.82	1; 58.82
*Staphylococcus*	2; 4547	0; 0	0; 0	2; 64.70
*Streptococcus*	22; 25,270	18; 43,005*	17; 23,257	17; 54,118^§^
*Veillonella*	2; 456	2; 611.1	1; 588.2	2; 117.6

Values are the number of positive samples; mean germ count

*p < 0.05 versus normal controls

^§^*p* < 0.05 versus preoperative

It can be observed that the average germ count increased after surgery and weight loss, albeit not significantly. In addition, *C. albicans* and non-albicans *Candida* species: *C. dubliniensis*, *C. kefyr*, and *C. lusitaniae* emerged after surgery, both in terms of the proportion of subjects and a significant germ count surge. The proportion of patients with *Neisseria* decreased significantly after surgery. A phenomenon of *Candida* only emerging where *Neisseria* was absent throughout or eliminated after surgery was also noticeable. Other genera affecting a considerably increased proportion of subjects upon weight loss included *Prevotella* (Table 3).

**Table 3:** Number and percentage of positive cultures for microorganisms in 17 surgery patients before and 6–12 months after bariatric surgery in decreasing order of pre-surgery values

	Pre-surgery no.	%	Post-surgery no.	%	*p* value
*Streptococcus*	17	100.0	17	100.0	N/A
*Neisseria*	10	58.8	4	23.5	0.0339
*Prevotella*	7	41.2	11	64.7	0.2482
*Fusobacterium*	2	11.8	3	17.6	0.6547
*Haemophilus*	2	11.8	4	23.5	0.4142
*Veillonella*	1	5.9	2	11.8	0.5637
*Granulicatella*	1	5.9	0	0.0	0.3173
*Rothia*	1	5.9	1	5.9	N/A
*Actinomyces*	1	5.9	0	0.0	0.3173
*Staphylococcus*	0	0.0	2	11.8	0.1573
*Candida*	0	0.0	5	29.4	0.0253
*Eikenella*	0	0.0	1	5.9	0.3173

## Discussion

In our study, no periodontal abnormalities were found either in the normal control or any of the obese groups. More and more studies suggest that excess weight is not a predisposing entity on its own, and periodontitis requires the simultaneous presence of other factors for its development. Saxlin et al. have also come to the conclusion that overweight and obesity are not substantial risk factors in the onset and pathogenesis of the periodontal disease (similarly, they studied a low-risk population) [10].

No periodontitis was observed after postoperative weight loss in our obese patients either. Similar results were obtained by Sales-Peres et al. in 2017: during their 1-year follow-up of bariatric surgery, probing depth and the extent of attachment loss did not change significantly from the preoperative baseline (although increased gum bleeding was observed during the first 6 months) [11].

Although periodontal status did not change remarkably after weight loss, changes did occur in the microflora of the crevicular fluid as the average germ count increased and new species such as non-albicans *Candida* species emerged. A number of studies suggest that changes to the quantitative and qualitative composition of the crevicular fluid are more common in patients undergoing bariatric surgery. In 2015, Sales-Peres et al. investigated the quantitative relations of 4 periodontal pathogenic bacterial species, *Porphyromonas gingivalis*, *Tannerella forsythia*, *Treponema denticola*, and *Prevotella intermedia*, prior to and 6 and 12 months after bariatric surgery. They found that the *P. gingivalis* count increased abruptly in the first 6 months and then decreased substantially; overall, the counts of other bacteria declined slightly over 12 months [7]. In contrast, of these bacteria, only *Prevotella* species were present in our study, and the proportion of subjects affected increased after weight loss.

Hashizume et al. investigated patients’ saliva sample levels of *Streptococcus*, *Lactobacillus* spp., and *C. albicans* before and 6 months after bariatric surgery. They detected high *C. albicans* levels both before and after the intervention and elevated *S. mutans* levels 6 months after bariatric surgery. However, they also reported that the patients with obesity in their study suffered from a number of chronic systemic comorbidities such as diabetes, for which they were treated with various medications and were prosthesis users, all of which predispose to the expansion of *C. albicans* [12]. None of these predisposing factors was present in our study, yet in addition to *C. albicans*, non-albicans *Candida* species such as *C. dubliniensis*, *C. kefyr*, and *C. lusitaniae* emerged after surgery. Typically, these non-albicans *Candida* species can be isolated from the oral cavity of immunosuppressed patients, mainly those on chemotherapy for tumors, and HIV-infected individuals [4, 5, 13, 14].

It was also observed in our study that the various *Candida* species only emerged where *Neisseria* species were absent throughout or eliminated after surgery. In 2017, Janus et al. investigated in vitro the effect of *C. albicans* on oral biofilm. In a similar fashion to our study, they found that biofilms without *C. albicans* contained more bacteria of aerobic and facultative anaerobic genera such as *Neisseria*, *Rothia*, and *Streptococcus* [15].

The strengths of our study include the fact that it provides a detailed picture of crevicular microflora changes following bariatric surgery. In contrast with previous research, our investigations were extended beyond bacteria to cover fungi as well, and we only included non-smoking subjects with good general health who had susceptibility factors neither for periodontal diseases nor for Candida infection. Nevertheless, our study has limitations: our findings are subject to more precise elucidation based on patient groups of greater sample sizes. In addition, the control groups could also be re-sampled to evaluate potential changes in crevicular fluid composition. Finally, a longer follow-up of several years could be undertaken to find out about the constant or variable nature of these results.

In conclusion, our study demonstrates that changes after bariatric surgery do affect the oral cavity and the periodontium as well; since the composition of crevicular gingival fluid changed 6 to 12 months after surgery, various non-albicans Candida species emerged, and the proportion of those affected by Prevotella increased. However, with an uninflamed periodontal condition before surgery, absence of predisposing factors, and good oral hygiene, periodontitis is highly unlikely to develop after surgery and weight loss even if changes to the crevicular microflora have taken place. For this reason, it would be important to assess the condition of the oral cavity and periodontium and to treat and eliminate possible inflammatory conditions in patients with obesity before surgery.
